# *Helicobacter pylori* Recombinant CagA Regulates Th1/Th2 Balance in a BALB/c Murine Model

**DOI:** 10.34172/apb.2020.031

**Published:** 2020-02-18

**Authors:** Nafiseh Paydarnia, Behzad Mansoori, Davoud Esmaeili, Tohid Kazemi, Mahyar Aghapour, Khalil Hajiasgharzadeh, Nazila Alizadeh, Behzad Baradaran

**Affiliations:** ^1^Immunology Research Center, Tabriz University of Medical Sciences, Tabriz, Iran.; ^2^Tabriz University of Medical Sciences, International Branch (Aras), Tabriz, Iran.; ^3^Student Research Committee, Tabriz University of Medical Sciences, Tabriz, Iran.; ^4^Department of Medical Microbiology, Baqiyatallah University of Medical Sciences, Tehran, Iran.; ^5^Department of Pathobiology, Faculty of Veterinary Medicine, University of Tabriz, Tabriz, Iran.

**Keywords:** *Helicobacter pylori*, Th1 immune response, Immunization, Lipopolysaccharide, Recombinant cytotoxinassociated gene A

## Abstract

***Purpose:*** *Helicobacter pylori* is recognized as one of the prevalent causes of human gastricinfection. In the present study, the role of mixed immunization with *H. pylori* lipopolysaccharide(LPS) and recombinant cytotoxin-associated gene A (rCagA) as a stimulator of host immuneresponses was determined.

***Methods:*** BALB/c mice were immunized with different formulations by the systemic administrationat 14-day intervals. The effects of the formulations plus CpG adjuvants were assessed before andpost-immunization in separated studies. Moreover, the expression of Th1/Th2 cytokines wasquantified in sera of immunized mice using reverse transcription polymerase chain reaction (RTPCR)test and the protein levels confirmed with enzyme linked immunosorbent assay (ELISA).Finally, the specific antibody levels in sera were studied by ELISA and the tendency of cellularresponse was examined by IgG1/IgG2a ratio.

***Results:*** Data of Western blotting verified the presence of constructed protein. Analysisof lymphocyte proliferation showed that CpG-conjugated rCagA increases lymphocytesproliferation compared to the control group. Also, it was shown that formulations containing LPSand rCagA promote a Th1 response indicated by interferon-gamma expression and induced Th1/Th2 balance. Additionally, the specific IgG1, total IgG and IgG2a levels elevated in response toall treatments. Ultimately, the IgG2a/IgG1 ratio in the mice immunized with rCagA-containingformulations increased.

***Conclusion:*** These results indicated that rCagA protein carried with CpG adjuvant not onlymaintained its antigenicity throughout the experiment but also induced robust Th1-biasedimmune responses. Therefore, it holds promise for the production of an efficient vaccine against *H. pylori* infection.

## Introduction

*Helicobacter pylori* is known for its well-characterized implication in gastritis, a widespread complication with high incidence. During the pathogenesis of gastritis, the gut epithelium completely loses its function as a result of repeated mucosal inflammation, which in turn disrupts the immune system function.^1^ It has also been indicated that *H. pylori*-induced gastritis has a critical etiological contribution to the progression of gastric cancer. *H. pylori* infection may represent a latent feature in many individuals that remained unrecognized for years.^2^ Mouse models have been extensively used to investigate the effectiveness and underlying mechanisms of vaccination. Numerous studies have focused on immunization routes to gain effective immunity. Though it is not identified that proper immunization routes can protect from *H. pylori*-induced diseases in individuals,^3^ it may be proved for human use in the future.^4^ Following *H. pylori*-induced infection, leukocytes and pro-inflammatory mediators recruit into the injured luminal surface. The strength of host inflammatory responses to *H. pylori* invasion is an indicative factor for mucosal damages, the less potent immunity the less protected gut epithelium.^5^ Lipopolysaccharide (LPS) is ab important surface antigen that represents an essential function in the stability of the bacterial outer membrane. LPS of *H. pylori* is alike to that in the other gram-negative bacteria,^6^ however, lipid A of*H. pylori* has been structurally separated from other Enterobacteriaceae LPS.^7^ LPS of *H. pylori* is identified by less and longer fatty acid residues, lack of 4-phosphate groups and an ethanolamine group bound to the glycosyl phosphate; therefore, *H. pylori* LPS exhibits weak biological functions by increased secretion of cytokines.^8^ Thus, the numerous activities of *H. pylori* LPS are more limited than other LPS such as those from *E. coli*.^5,9-12^ The pathophysiologic conditions such as apoptosis of luminal cells and gastritis can be triggered by *H. pylori* infection.^13^ The cytotoxin-associated gene A (CagA) protein acts as an oncoprotein and virulence factor for *H. pylori*, which alters intracellular signaling pathways (such as SHP-2, PAR1/MARK kinase and canonical Wnt/β-catenin) and weakens the cell-cell junctions in the mammalian gastric epithelium.^14-16^ CpG oligodeoxynucleotides are known to exert T helper1 (Th1)-based immune responses and have been successfully used as vaccine adjuvants in several trials.^17^ Multicomponent-subunit vaccines have several advantages such as a good safety profile, high efficacy and appropriateness for manufacturing in industrial processing.^18^ Several studies have already demonstrated that vaccination with a combination of recombinant *H. pylori* subunit antigens and various adjuvants can potentially induce appropriate immune protection versus *H. pylori* infection.^19,20^ The main purpose of the current experiment is to evaluate the potency of multi-component formulation composed of *H. pylori* rCagA and LPS antigens with CpG adjuvant for induction of immunity versus *H. pylori* in BALB/c mice.

**Table 1:** Primer sequences used for reverse transcription polymerase chain reaction (RT-PCR) analysis

**Gene**	**Reverse primer (5ʹ–3ʹ)** **Forward primer (5ʹ–3ʹ)**	**Amplicon size (Bp)**	**Tm**
IL-12	F 5' ATGATGACCCTGTGCCTTGG 3' R 5' CACCCTGTTGATGGTCACGA 3'	282	55
IFN-γ	F 5' ACTGGCAAAAGGATGGTGAC 3' R 5' TGAGCTCATTGAATGCTTGG 3'	237	54
IL-10	F 5' CCAAGCCTTATCGGAAATGA 3' R 5' TTTTCACAGGGGAGAAATCG 3'	162	55
IL-4	F 5' GCCTGCTTTTCACATGAGGT 3' R 5' AAATATGCGAAGCACCTTGG 3'	250	54
β-actin	F 5' AGCCATGTACGTAGCCATCC 3' R 5' CTCTCAGCTGTGGTGGTGAA 3'	228	54

IL-12: interleukin 12, IFN-γ: interferon γ, IL-10: interleukin 10, IL-4: interleukin 4, Tm: melting temperature, Bp: base pair

## Materials and Methods

### Extraction and characterization of *H. pylori* LPS

Bacterial colonies collected from gastric biopsy samples were sonicated, and then extraction of *H. pylori* LPS was carried out by Westphal method. The sonicated bacterial solutions were added to the same volume of hot phenol-water (9:1, V:V) and then the samples were shaken for half an hour at 65-70 rpm. After centrifugation at 3500 rpm/30 min in 4°C, aqueous phases were collected (this step was repeated three times). All collected phenolic phases were dialyzed against distilled water for 48 hours for the elimination of phenol (pH = 7.4). The extracted LPS were concentrated to 1/5 of the initial volume and then digested with the use of RNaseH and DNase I (Sigma, St. Louis, MO, USA) enzymes with a final volume of 50 μg/mL at 37°C at 4 hours.^8^ The digested extract was washed in hot water for 15 minutes and then placed at 4°C overnight. Then, the supernatants were centrifuged at 3000 rpm/min for 30 minutes. After that, the mixture was dialyzed against distilled water for 48-72 hours. By centrifugation at 5000 rpm/min for 30 minutes, precipitates were collected, then washed and dialyzed against distilled water for 48 hours. The LPS extracts were collected by centrifugation at 100 000 g for 2 hours and pellets were dialyzed in distilled water then were lyophilized.^8^

### Production of *H. pylori* rCagA protein

A primer was designed to amplify *H. pylori* CagA gene target fragment according to the available sequences in the GeneBank. The primers had a *Bam* HI site incorporated into the 5’ end and a *Sac* I site at the 3’ end and their sequences are as follows: F: 5’- AAGGATCCACTAACGAAACCATTGACCA-3’ and R: 5’-AAGAGCTCACTCCCTCAACTCTAACATT-3’ that enable amplification of a fragment tolength of nearly 841 bp. Escherichia coli DH5 ± and *E. coli* BL21were applied as cloning and expression prokaryotichosts. The pJET1.2 (Fermentas, Ontario, Canada) and pET28a (Novagen, San Diego, CA) were exploited for cloning and expressionof the target open reading frame. A primer pair was designed to amplify the terminal 841 bp of the coding region of CagA.The rCagA proteins collected by centrifugation at 14 000 g for 20 minutes and then collected. Purified rCagAwas also subjected to Western blot analysis.

### Animals

All the animal studies have been conducted according to relevant national and international guidelines and Institutional Animal Care and Use Committee (IACUC) of Tabriz University of Medical Sciences. Healthy male BALB/c mice (15-20 g; 6-8 weeks old, purchased from Iran Pasteur Institute) were adapted and randomly distributed into four experimental groups (n = 10/each group) and housed in the laboratory under standard animal room conditions (12-hour dark/light cycles at 23-25°C). The animals were given access to food and water ad libitum. The randomization procedure was performed using a random number table, in which each animal was marked with a specific number and randomized into four experiment groups based on the arbitrary numbers on the table.

### Lymphocyte proliferation test

The mice were force-fed three times followed by two intramuscular boosters. Two weeks following the latest immunization, spleens from immunized and non-immunized mice were excised and suspended in cold phosphate-buffered saline (PBS) at 4°C under sterile condition; red blood cells were lysed using NH4Cl buffer. The cell suspension was prepared in complete RPMI 1640 (Gibco, BRL, Grand Island, NY, USA) and adjusted to 5×10^6^ cells per milliliters. 100 μL of cell suspension was added to each well of flat-bottomed 96 well plates and rCagA at the concentration of 1 μg/mL was added to each one. Some wells have not added an antigen, which considered as negative controls while phytohemagglutinin (PHA) (Gibco, BRL, Grand Island, NY, USA) used as a positive control at a final concentration 5 μg/mL. The volume of all wells adjusted to 200 μL. Following incubation for 72 h at 37°C in 5% CO2 humidified incubator, cell proliferation was measured by using 3(4,5-dimethylthiazol-2-yl)-2,5-diphenyltetrazolium bromide; thiazolyl-blue (MTT) dye assay. Briefly, 20 μL MTT (5 mg/mL) (Sigma, St. Louis, Mo, USA) was added and plates were further incubated at 37°C for 4 hours. Subsequently, the plates were centrifuged at 300 gfor 10 minutes and after removal of the media, 200 μL dimethyl sulfoxide (DMSO) was added to the wells. The values of optical density (OD) of the cells were evaluated at 570 nm with an ELISA Reader (Sunrise RC, Tecan, Switzerland). Stimulation index (SI) was determined according to the following formula; SI= OD of the wells incubated with antigen/OD of the wells without antigen exposure.^9,12^

### Immunizations and experimental design

All groups were immunized three times orally and subsequently two times intramuscularly (IM) at 14-day intervals. Immunizations were conducted with rCagA (5 μg) + CpG (15 μg) (RC), LPS (20 μg) + CpG (15 μg) (LC), rCagA (5 μg) + LPS (20 μg) + CpG (15 μg) (RLC). The optimal doses for immunization were selected based on the results of mouse lethality test and MTT. Control animals were received PBS using the same approach.

### RT-PCR for evaluation of cytokine expression

Total RNA was only extracted from the isolated lymphocyte in peripheral blood of RLC-immunized mice and complementary DNA was synthesized. In brief, Total RNA was isolated from the cells using TRIzol (Riboex, Gene All Biotechnology, Seoul, Korea). The total RNA purity and integrity was confirmed by using a NanoDrop (Thermo Scientific, USA). Then 1μg total RNA was reverse transcribed into cDNA (Biofact, South Korea). To further confirm the capability of rCagA-contained formulation for triggering cellular and humoral immunity, reverse transcription polymerase chain reaction (RT-PCR) was performed for interferon-gamma (IFN-γ), IL-12, IL-10 and IL-4 cytokines and the intensity of each band was estimated by densitometry and normalized against β-actin expression as a housekeeping gene. Primers for the corresponding genes are presented in Table 1.

### Antibody measurement

Enzyme-linked immunosorbent assay (ELISA) technique was used for antibody measurement. Sera were collected 10 days following the immunization and the concentration of IgG1, total IgG and IgG2a antibodies specific to *H. pylori* antigens and also IgG2α/IgG1 ratio were measured. Briefly, 96-well plates were coated with ovalbumin (Sigma, St. Louis, MO, USA) (150 μg/well) in carbonate buffer, (pH 9.6), and then incubated overnight at 4°C. Serum samples were diluted with 200 μL of PBS at 37°C for 1 hour, followed by incubation of a peroxidase conjugated anti-mouse IgG and IgM (Sigma, St. Louis, MO, USA), and chromogenic substrate (o-phenylenediamine in 10 mL citrate buffer (pH 5.0) plus 0.02% H2O2. The absorbance was determined at 490 nm using a Stat Fax 2100 plate reader (Awareness Technology Inc, Palm City, FL, USA).

### Statistical analysis

All results were presented as mean ± SD. Statistical analysis was done through one-way ANOVA by GraphPad Prism 6.0 (GraphPad Inc, CA, USA). Multiple comparisons between control and test groups were corrected using post hoc Bonferroni’s correction. Tukey’s multiple comparison test was used for statistical correction of IgG2a/IgG1 ratio in first and last immunization of each group. *P* value < 0.05 were considered statistically significant.

## Results and Discussion

### Construction of *H. pylori* recombinant CagA

**Figure 1:**
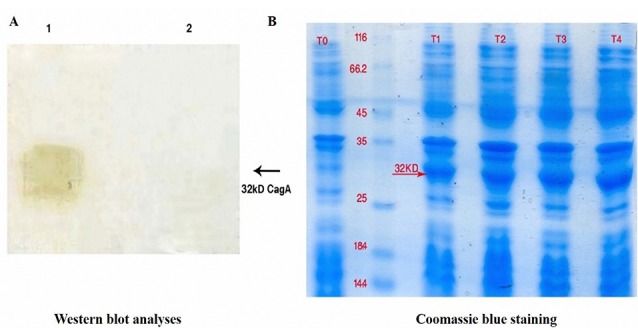
(A) Confirmation of recombinant cytotoxin-associated gene A (rCagA) protein. Western blot analyses evidenced the presence of *E. coli* BL21 (DE3) expressing *H. pylori* CagA protein (lane 1) in comparison with mock control (lane 2). (B) Coomassie blue staining confirmed the production of recombinant *E. coli* BL21 *H. pylori* CagA (T1, T2, T3 and T4) contrary to non-induced *E. coli* BL21 (T0).

Although vaccination as a humoral arm of immunity attracted considerable attention for preventing negative outcomes of *H. pylori* infection, to date, no remarkable breakthrough has been observed. The immune-protective effects are restricted by the efficiency of the selected vaccine antigens and the choice of vaccination route. For example, Rossi et al have reported that IM vaccination with three recombinant *H. pylori* antigens, namely CagA, VacA, and NAP can decrease bacterial colonization in beagle dogs.^21^ Additionally, it has been shown that a combination of multiple antigens, each being involved in a different function, e.g. colonization and pathogenesis, could provide a better immunity against*H. pylori*. Another study has also indicated that immunization with urease B (UreB), HspA or HpaA can protect animals against *H. pylori* challenging.^19^ Adjuvants are usually used in recombinant vaccine formulations to enhance the immune effect.^10-12^ In recent experiments, mucosal adjuvants have found extensive application in the induction of immunity against *H. pylori*. For instance, the cholera toxin and the heat-labile *E. coli* enterotoxin have proved to be effective mucosal adjutants combined with *H. pylori* inactivated whole-cell or recombinant antigens.^21^ However, since both adjuvants are toxic in humans, CpG has been produced to reduce the toxicity and develop Th1 response.^9^ CpG oligonucleotides contain unmethylated cytosine-guanosine dinucleotide motifs comparable to those observed in bacterial DNA that trigger Toll-like receptor 9 (TLR9) activation in the mammalian immune cells lead to Th1-tilted response.^22^ The *H. pylori* rCagA gene was subcloned into pET28a and then recombinant plasmid pET28a- rCagA was electroporated into *Escherichia coli* BL21 (DE3) to form recombinant *E. coli* BL21 (DE3) pET28a-*r* CagA. The expressed *H. pylori* rCagA was assessed by Western blot analyses (Figure 1A) and Coomassie blue staining (Figure 1B). We found that the *H. pylori* rCagA protein was expressed by recombinant *E.coli* BL21 (DE3) pET28a-*r* CagA strains (lane 1), while *E. coli* BL21 (DE3) pET28a alone did not show any detectable *H. pylori* rCagA (lane 2) (Figure 1A).

### Proliferative responses to purified antigens

**Figure 2:**
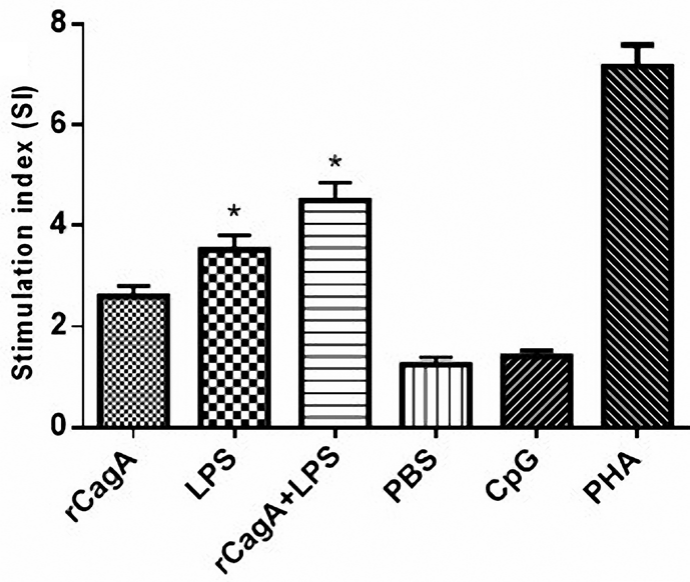
The proliferation of splenocytes isolated from BALB/c mice immunized with the recombinant cytotoxin-associated gene A (rCagA), lipopolysaccharide (LPS), LPS-rCagA conjugate, phosphate-buffered saline (PBS), CpG oligodeoxynucleotides and Phytohemagglutinin (PHA) respectively. As shown the LPS-rCagA combination increased the stimulation index (SI) and consequently the growth of splenocytes.

Splenocytes from animals immunized with the rCagA protein proliferated in response to the specific antigens. The rCagA/LPS combination seems to markedly increase the growth of cultured splenocytes. In addition, all cell groups responded to the PHA as a positive control of proliferation. The pattern of the proliferative response of splenocytes has been illustrated in Figure 2.

Typically, the innate immunity is responsible for early detection and reaction to the invaded pathogens. Activation of CD4^+^ T cells by MHC II-linked antigens is crucial for the protection against *H. pylori* infections.^23^ It has been known that *H. pylori* instigates a Th1-skewed response and also as a pathogenic microorganism was anticipated to exert a moderate Th2-related balancing activity.^17^ While, the immune system is neither able to clear off, nor prevent recurrence of *H. pylori* infection. It has been addressed that the un-phosphorylated CagA dislocates the integrity of epithelial cells and initiate an inflammatory response.^22^ Also, *H. pylori’s* rCagA protein has been shown to enhance the immunogenicity of peptide and polysaccharide vaccines.^24^ Our findings further reveal that immunization of mice with rCagA-contained antigens stimulated cellular and humoral responses in mice.

### Th1/Th2 cytokine pattern in PBMC and serum

**Figure 3:**
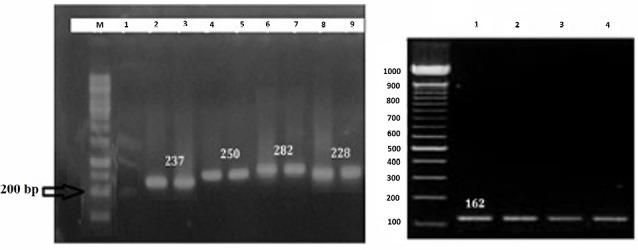
(A) Gel electrophoresis containing cytokine gene bands pre- and post-immunization with rCagA+LPS+CpG (RLC). Lane M: 100-bp DNA marker, Lane 1: IFNγ gene band before immunization with RLC, Lanes 2 and 3: IFNγ gene band post-immunization with RLC, Lanes 4 and 5: IL-4 gene band post-immunization with RLC, the Lanes 6 and 7: IL-12 gene band post-immunization with RLC, Lane 8: β-actin gene band before immunization and Lane 9: β-actin gene band post-immunization as a reference gene. (B). The expression of IL-10 pre- (Lane 1-2) and post-immunization (Lane 3-4) with RLC.

Results of RT-PCR demonstrated that the intensity of Interferon gamma (IFN-γ) and interleukin (IL)-10 bands were increased in the RLC-immunized group as opposed to the control group. On the other hand, β-actin expression as a housekeeping gene, showed no significant difference after and before immunization (Figure 3).

**Figure 4:**
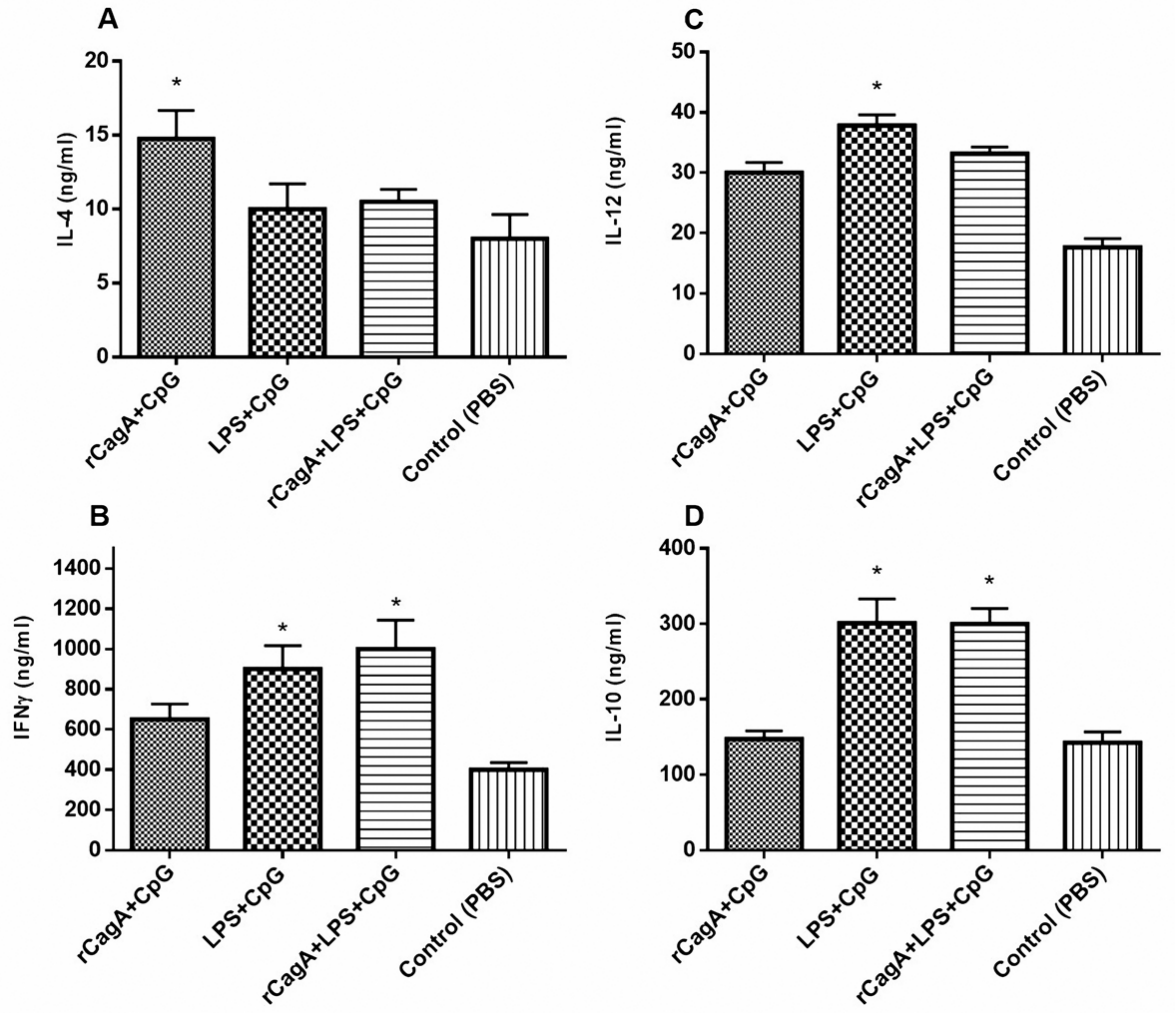
Production of Th1 and Th2 cytokines in response to the formulations. The post-transcriptional levels of interleukin (IL)-4 (A), IL-10 (B), IL-12 (C) and Interferon-gamma (IFN-γ) (D). *P* value < 0.05 considered as a significant difference in comparison with the PBS-receiving group.

These data also showed that following 4h incubation the highest level of IFNγ and IL-10 mRNA expression was observed and this response remained until the next 24 hours. Then it falls to the baseline level. To further elucidate antigen-specific Th1/Th2 response profile, the protein level of IL-4, IL-10, IL-12 and IFN-γ were quantified by commercial ELISA kit. As given in Figure 4, the post-transcriptional level of IFN-γ and IL-12 cytokines increased in all immunized groups, suggesting Th1 immune response. While a higher level of Th2 cytokines was observed in LPS-contained groups (RLC and LC) (Figure 4A-D).

Th1 cells are activated in the presence of intracellular pathogens and regulate the cellular immune functions via the production of pro-inflammatory cytokines, including IFN-γ, and IL-2. On the other side, Th2 response is activated in the presence of extracellular pathogens, mediating the humoral immune response, characterized by the release of distinct cytokines such as IL-4, IL-5, IL-6, IL-10 and IL-13.^22^ Meanwhile, *H. pylori* activated both Th1-Th2 responses, leading to the stimulation of antibody-producing B lymphocytes associated with Th1 response such as IgG2a and IgA and those contributed to the Th2-specific antibodies as IgG1. It has been well-known that *H. pylori* infection disrupts Th1 immune response, therefore modulating Th1 response would be of utmost therapeutical interest.^17^ Although earlier studies showed a protective role for Th2 response in reducing *H. pylori*-induced gastritis, more recent reports suggested that protection against *H. pylori* is correlated with the progress of Th1 responses, which accompanied with high levels of IFN-γ and IL-12 and elevated IgG2a/IgG1 ratio as well.^16,21,23,25^ We illustrated that the concomitant treatment of rCagA and LPS accompanying with CpG have synergistic outcomes and enhances a Th1 immune reaction (IFN-γ, IL-12, IgG2a) that aid in the protection of *H. pylori* infection.

The balance between Th1/Th2 responses is crucial for *H. pylori* clearance. The continuous *H. pylori* infection normally results in Th1-polarized responses, while successful *H. pylori* immunization could result in a balanced Th1/Th2 response.^26^ The comparative results unveil that formulations containing rCagA promote a Th1 response indicated by IFN-γ and induced Th1/Th2 balance through elevating IL-4, IL-10 and TGF-β levels which were in concurrence with the increased proliferation of Spleen cells. In this experiment, we demonstrated that *H. pylori* multi-component formulations could stimulate immune responses in mice by an Oral/IM immunization procedure, which may be used as the effective vaccination regimen against *H. pylori*-associated gastric infection. We showed that our 32 kDa designed rCagA proteins from amino ends of *H. pylori* are immunogenic and enable to trigger proper Th1 immune responses in mice when carried by CpG adjuvant. Our findings suggest that immunization with rCagA-containing formulations promotes a Th1 immune response, indicating proper induction of humoral and cellular responses. *H. pylori* rCagA serves as an appropriate antigen that can efficiently promote the production of *H. pylori*-specific serum immunoglobulin, as evaluated by ELISA.

### *Helicobacter pylori* specific antibody responses

**Figure 5:**
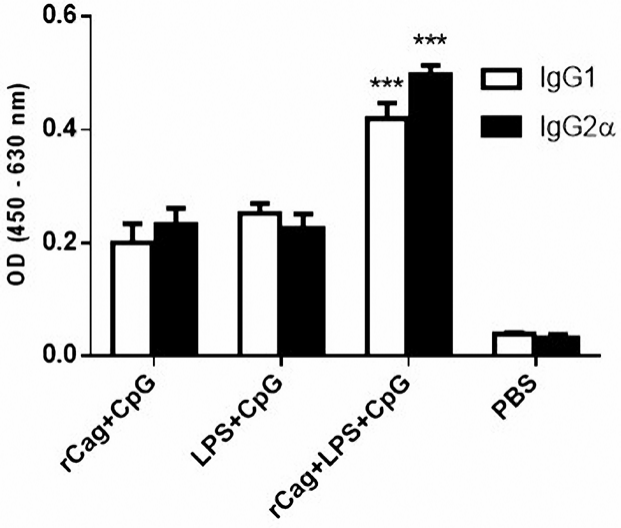
The IgG2α/IgG1 ratio in immunized groups. The IgG1 and IgG2α levels were studied in different groups of mice as measured by ELISA technique. The IgG2a/IgG1 ratio in the rCagA+LPS+CpG (RLC) receiving groups was increased. This ratio was more than 1 suggesting the capability of these formulations to polarize Th1 immune response. The RLC formulation provokes the most powerful immune response.

**Figure 6:**
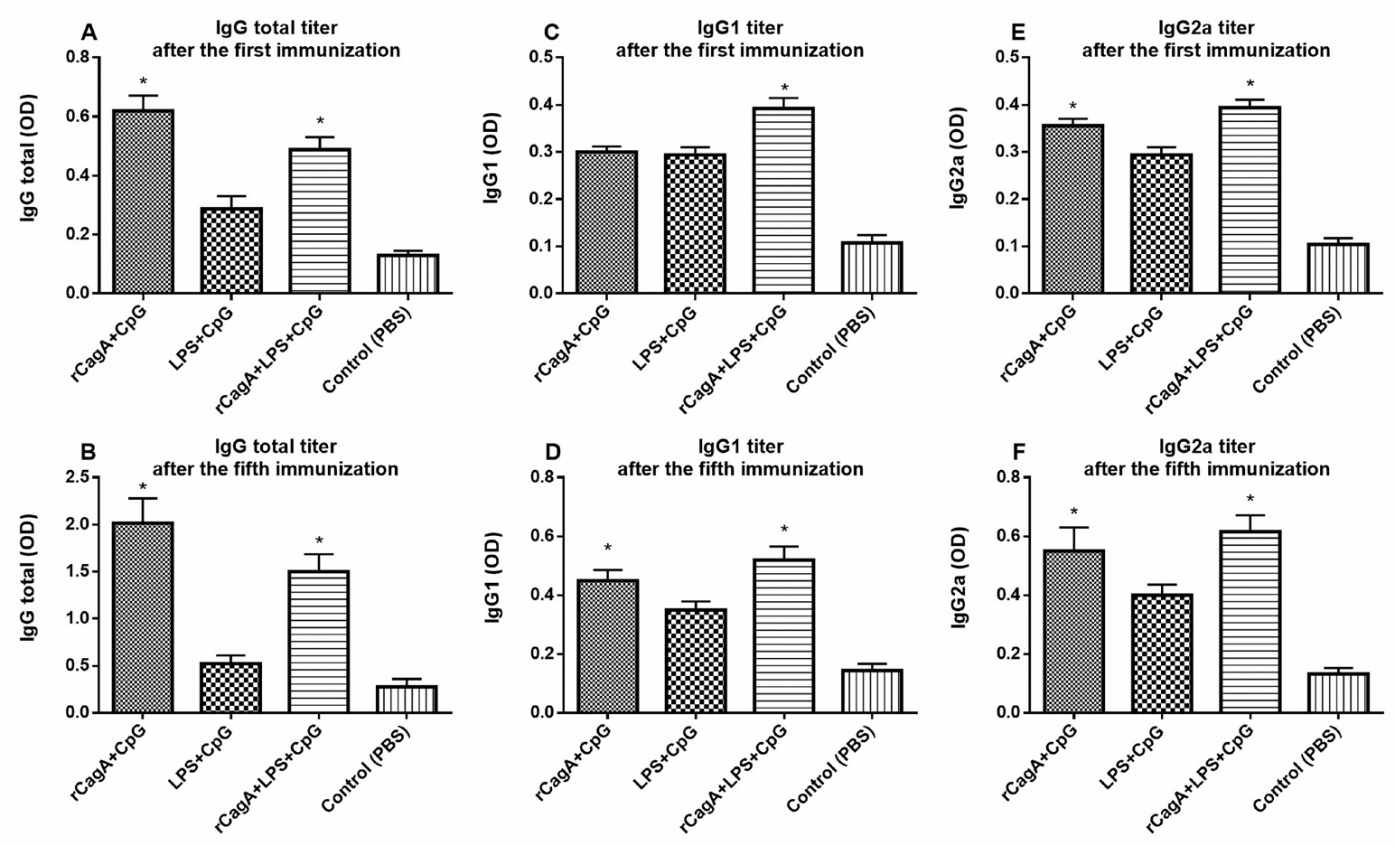
IgG total (A, B), IgG1 (C, D) and IgG2a (E, F) responses against specific antigens after the first and the fifth immunizations, respectively. Sera from four mice per group collected immediately after the immunization and individually examined by enzyme linked immunosorbent assay.

*Helicobacter pylori* specific antibodies, including IgG1, IgG2a and total IgG were measured in immunized mice serum by ELISA. The IgG2a/IgG1 ratio in rCagA-receiving groups was increased, indicating Th1 type immune response (Figure 5). As has been illustrated, the total IgG response against a specific antigen was gradually elevated in mice administrated with RC and RLC throughout the immunization experiment (*P* < 0.05). Immunization with RC elicited most powerful specific IgG response, whereas immunization with LC acted conversely and induced lower levels of antibodies in mice sera. As shown in Figure 6, RLC immunization regularly elicited high levels of Ig2a as well as IgG1 antibodies with repeated systemic challenging.

## Conclusion

In conclusion, we propose that immunization with appropriate *H. pylori*-extracted antigens and carrying them with an adjuvant can stimulate immune responses. The pathogenicity of *H. pylori* depends on the generation of numerous virulence factors. As one of the most well-known examples is CagA. After the delivery of CagA into the cytosol of gastric epithelial cells, it becomes tyrosine-phosphorylated and imitates a host cell protein in coupling and activation of various signaling factors.^27^ This oncoprotein remains the only recognized mechanism that is translocated by the T4SS of *H. pylori* into gastric epithelial cells where it causes to the activation of the host cell kinases and consequently activates numerous signal transduction pathways.^27^ Previously published researches disclosed that the protective effect of double-antigen vaccines is more specific than a single-component vaccine.^25,28,29^ Overall, RLC formulation can serve as a promising candidate for the fight against the *H. pylori* infection and possibly provide a prophylactic tool to reduce the progressive growth of *H. pylori-* associated gastric cancers. However, future efforts should focus on exploring the immune-protective effect of RLC formulation in *H. pylori*-induced animal model of gastritis. Altogether,this preliminary study showed that immunization with RLC in a murine model is capable of balancing Th1/Th2 responses. As the most important limitations of this study, we can point out to the uncertainty of all the antigens that cause to *H pylori*-induced immune responses and the extended variety of immune-inducing factors as well as the limitation of examining all these factors. But based on the results of this research, we suggest RLC formulation as a promising prophylactic candidate for *H. pylori*-induced infection.

## Ethical Issues

All experiments and procedures were conducted in compliance with the ethical principles of Tabriz University of Medical Science, Tabriz, Iran and approved by the regional ethical committee for medical research.

## Conflict of Interest

Authors declare no conflict of interest in this study.

## Acknowledgments

The authors want to thank Tabriz University of Medical Sciences for support to conduct this research.
